# Prognostic Factors for Visual Outcomes in Closed Idiopathic Macular Holes after Vitrectomy: Outcomes at 4 Years in a Monocentric Study

**DOI:** 10.1155/2022/1553719

**Published:** 2022-04-23

**Authors:** Alexandre Lachance, Mélanie Hébert, Eunice You, Jean-Philippe Rozon, Serge Bourgault, Mathieu Caissie, Éric Tourville, Ali Dirani

**Affiliations:** ^1^Faculté de Médecine, Université Laval, Québec, QC, Canada; ^2^Département d'ophtalmologie et d'oto-rhino-laryngologie–chirurgie cervico-faciale, Centre Universitaire d'Ophtalmologie, Hôpital du Saint-Sacrement, CHU de Québec-Université Laval, Québec, QC, Canada

## Abstract

**Purpose:**

To identify predictive factors of visual outcomes in the eyes after successful macular hole (MH) surgery.

**Methods:**

It is a retrospective monocentric study of the eyes that underwent successful vitrectomy for full-thickness MH in an academic, tertiary care center (CHU de Québec–Université Laval, Québec, Canada) between 2014 and 2018. We included a single eye per patient and excluded the eyes with ocular comorbidities. Clinical and anatomical features of patients were collected, including demographics, MH duration, baseline MH size, baseline visual acuity (VA), and final VA. Multiple logistic regressions were performed to determine predictive factors of VA ≥70 ETDRS letters (Snellen equivalent: 20/40) and VA gain ≥15 ETDRS letters at final follow-up. Areas under the receiver operating characteristic curve (AUC) were used to determine the performance of each model and identify the Youden index maximizing performance at a given threshold.

**Results:**

A total of 460 eyes were included in this study; 274/460 eyes (60%) achieved final VA ≥70 ETDRS letters and 304/460 eyes (66%) had a VA gain ≥15 ETDRS letters at 24 months follow-up. Multiple logistic regression analyses showed that the main predictive factors for final VA ≥70 ETDRS letters (model AUC = 0.716) were baseline VA (OR = 1.064; *p* < 0.001), MH duration (OR = 0.950; *p*=0.005), and age (OR = 0.954; *p*=0.004). Predictors of VA gain ≥15 ETDRS letters at final follow-up (model AUC = 0.615) were baseline VA (OR = 0.878; *p* < 0.001), MH duration (OR = 0.940; *p* < 0.001), and MH size (OR = 0.998; *p*=0.036). Thresholds for the final VA ≥70 ETDRS letters model and the VA gain ≥15 ETDRS letters model were VA ≥55.5 ETDRS letters (Snellen equivalent: 6/30) and MH size of 237 *μ*m, respectively.

**Conclusion:**

The eyes with shorter MH duration, smaller MH size, and higher preoperative VA achieved better visual outcomes after successful MH surgery.

## 1. Introduction

Optical coherence tomography (OCT) and wide field retinography contributed to the change of the practice of physicians around the world and helped them to proper diagnose, monitor, and treat numerous eye diseases as macular hole (MH) [1, 2].

Idiopathic full-thickness MH affects 1 in 250 people and results in significant visual impairment, including reduced visual acuity (VA) and metamorphopsia [3]. Recent studies have reported MH closure rates after a first surgical procedure between 78% and 96% [4–6]. However, anatomical closure does not necessarily translate into visual improvement. In a recent study by Essex et al., final VA was less than 70 Early Treatment of Diabetic Retinopathy Study (ETDRS) letters and improved less than 15 ETDRS letters at 24 months in a third of patients successfully operated for MH [7]. This highlights the importance of assessing visual outcomes in addition to anatomical outcomes.

Previous studies have tried to identify prognostic factors of visual outcomes after successful MH surgery, but the impact of many of these factors remains unclear. Moreover, these studies were multicentric, increasing surgical heterogeneity, and they were limited by small sample sizes and short follow-up periods, which may underestimate final visual outcomes [5, 7, 8].

The aim of this study is to identify predictive factors for visual outcomes at long-term follow-up in the eyes with successful MH closure after vitrectomy.

## 2. Methods

### 2.1. Study Design and Setting

All consecutive patients operated for idiopathic full-thickness MH between 2014 and 2018 at the Center Hospitalier Universitaire de Québec–Université Laval (Canada) were identified. The eyes were operated on by one of five vitreoretinal surgeons. Patient records were systematically reviewed to identify patients with successful MH closure after primary vitrectomy retrospectively. The primary outcome was final VA ≥70 ETDRS letters (Snellen equivalent: 20/40). The secondary outcome was VA gain ≥15 ETDRS letters by final follow-up. These thresholds were chosen as a VA gain of ≥15 ETDRS letters is considered to represent a clinically significant improvement and has also been shown to correlate with a clinically meaningful improvement in patient-perceived outcomes, whereas achievement of ≥70 ETDRS letters has important quality of life implications, including driving [9].

This study was approved by the Institutional Review Board of the Center Hospitalier Universitaire de Québec–Université Laval (2021-5371), and adheres to the tenets of the Declaration of Helsinki.

### 2.2. Eligibility Criteria

We reviewed the record of all eyes operated for MH with a pars plana vitrectomy with internal limiting membrane (ILM) peeling using 0.06% trypan blue (TB) dye (VisionBlue™, DORC, Zuidland, Netherlands) or 0.05% diluted indocyanine green (ICG) dye (ICG, Diagnostic Green GmbH, Aschheim-Dornach, Germany) and gas or air tamponade. The type of tamponade used was at the discretion of the surgeon. All patients had been advised to position face-down after surgery for 5–7 days. Only patients with an anatomic MH closure confirmed by spectral domain OCT following surgery were included. Exclusion criteria included patients with a follow-up of less than four weeks, history of vitrectomy for any reason, and intraoperative use of a silicone oil tamponade or special techniques (e.g., free flap, inverted flap, and retinal autografts). The eyes with stage 1 MH, lamellar MH, and MH secondary to other causes (e.g., trauma, age-related macular degeneration (AMD), type 2 macular telangiectasia, and retinal detachment) and eyes with ocular comorbidities that could potentially affect VA including high refractive or axial myopia (i.e., ≥6 diopters of myopia or axial length ≥26 millimeters) were excluded. In patients with bilateral MH on initial presentation, only the first eye operated was included.

### 2.3. Data Extraction

The medical records of all patients were systematically reviewed by the main investigator (AL). Preoperative data collected included age, sex, lens status, myopia, MH duration (defined in our study as duration between the first reference and the time of surgery [10]), baseline VA, and MH size on initial presentation. Operative data included the surgical technique (i.e., vitrectomy or combined phacovitrectomy), type of dye, and tamponade. Postoperative data included VA at 2 weeks, 3 months, 6 months, 12 months, 24 months, 36 months, and 48 months postoperatively. Lens status was recorded at each visit.

VA was originally recorded on Snellen chart and were converted to ETDRS letters for analysis [11]. Counting fingers was recorded as 10 ETDRS letters. We had no patients with VA of hand motion, light perception, or no light perception.

All OCT scans were performed using the CIRRUS HD-OCT 5000 machine (Carl Zeiss Meditec, Jena, Germany). We evaluated preoperative OCT of all patients included in the study for MH size (measured as the minimum hole width or the narrowest aperture size in the middle retina, as defined by the Vitreomacular Traction Study Group [12]), presence of cystic cavities (defined as the intraretinal space in the foveal wall of the MH), and presence of elevated MH edges (defined as the presence of elevated edges of neurosensory retina in relation to the retinal pigment epithelial plane). We measured the diameter of MH as defined by the Vitreomacular Traction Study Group [12] and not as the base diameter and height of the wall, since this is more commonly used clinically.

### 2.4. Statistical Analysis

Data are presented as mean ± standard deviation for continuous variables and as frequencies (percentages) for categorical variables. Characteristics and variables were compared between the two final VA groups (i.e., <70 ETDRS and ≥70 ETDRS letters) and between the two final VA gain groups (i.e., <15 ETDRS and ≥15 ETDRS letters). We used independent Student's *t*-test or Mann–Whitney *U* test as appropriate for continuous variables and chi-square analysis for categorical variables. Paired *t*-tests were used to compare continuous variables in the same patient across timepoints. The Shapiro–Wilk test and Q-Q plots with 95% confidence intervals were used to test for normal distribution of continuous variables. Collinearity between variables was assessed using Spearman's rank correlation coefficients.

A multiple logistic regression model was built to identify predictive factors of VA ≥70 ETDRS letters and VA gain ≥15 ETDRS letters at final follow-up. These included age, sex, baseline BCVA, MH duration, MH size, tamponade agent used, dye used, bilateral disease, presence of preoperative cystic cavities, presence of preoperative elevated edges of MH, follow-up duration, and pseudophakia at final follow-up. A backwards elimination strategy was used to manually select variables from the full model, with variables *p* > 0.2 removed. Odds ratios (OR) and 95% confidence interval (CI) were calculated for each variable in the final model. All final models were adjusted for age. Baseline BCVA, MH duration, and MH size were also adjusted for in the final models to identify factors most associated with final VA. For the outcome VA ≥70 ETDRS letters, lens status was summarized using pseudophakia at final follow-up to account for patients who were pseudophakic at baseline and patients who had phacoemulsification during or after MH surgery. For the VA gain outcome, lens status included a variable for pseudophakia at baseline and for phacoemulsification with intraocular lens implantation during or after MH surgery.

Receiver operating characteristic (ROC) curves were used to analyze thresholds in predictive factors for VA ≥70 ETDRS letters and VA gain ≥15 ETDRS letters at final follow-up. The Youden index maximizing sensitivity and specificity for the outcomes are reported along with areas under the ROC curve (AUC).

Statistical analyses were performed using *R* for Windows (version 3.6.3; *R* Foundation for Statistical Computing) and IBM SPSS Statistics for Windows (version 25.0; IBM Corp., Armonk, NY). Statistical significance was set at *α* = 0.05.

## 3. Results

A total of 460 eyes were included in the study. The mean age was 69 ± 8 years. Of these, 316 (69%) were women and 113 (25%) were pseudophakic eyes. In total, 274/460 eyes (60%) achieved final VA ≥70 ETDRS letters and 304/460 eyes (66%) had a VA gain ≥15 ETDRS letters at 24 months follow-up.  Table 1 and Table 2 present baseline, intraoperative, and postoperative characteristics for the former and the latter outcomes, respectively. Pars plana vitrectomy with removal of posterior hyaloid, ILM peeling (after dye usage), and gas or air tamponade was performed in all eyes. Combined phacoemulsification was carried out in only 2 cases.

We assessed baseline characteristics associated with final VA ≥70 ETDRS letters using univariate analysis (Table 1). The multiple logistic regression analysis (Table 3) showed that younger age, higher baseline VA, shorter MH duration, MH elevated edge on preoperative OCT, the use of TB dye, pseudophakia at final follow-up visit, and longer duration of follow-up were independent predictors of final VA ≥70 ETDRS letters (all *p* < 0.05). The variable MH size was not an independent predictor of final VA ≥70 ETDRS letters in our study (OR: 0.999, 95% CI: 0.997–1.000; *p*=0.140) but was collinear with baseline VA (Spearman's coefficient correlation of −0.568; *p* < 0.001). Thus, patients with smaller MH size tended to have a better baseline VA.

We also assessed baseline characteristics associated with VA gain ≥15 ETDRS letters using univariate analysis (Table 2). The multiple logistic regression analysis (Table 4) for predictors of VA gain ≥15 ETDRS letters showed that the independent predictors were worse baseline VA, smaller MH size, shorter MH duration, preoperative pseudophakia, and combined phacovitrectomy/phacoemulsification postvitrectomy (all *p* < 0.05).

Baseline VA, MH duration, and MH size were the three main prognostic factors found for the two functional outcomes. Higher baseline VA resulted in a better final VA result, while worse baseline vision was a predictor of better VA gain. Moreover, shorter MH duration and smaller MH size were predictors of better final VA and VA gain. However, smaller MH was not associated with VA gain in univariate analysis; rather, the opposite effect was observed. In our study, younger age, MH elevated edge on preoperative OCT, the use of TB dye, and longer follow-up duration were significant predictors of final VA but not of VA gain ≥15 ETDRS letters.

### 3.1. Visual Acuity Improvement

**Figure 1:**
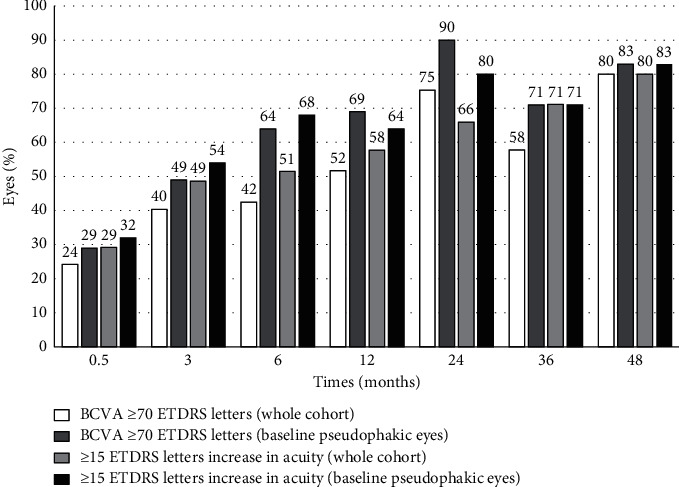
Long-term visual results after MH surgery for final VA ≥70 ETDRS letters and VA gain ≥15 ETDRS letters. BCVA ≥70 ETDRS letters (whole cohort). BCVA ≥70 ETDRS letters (baseline pseudophakic eyes). ≥15 ETDRS letters increase in acuity (whole cohort). ≥15 ETDRS letters increase in acuity (baseline pseudophakic eyes).

Mean VA improvement at 2 weeks, 3 months, 6 months, 12 months, 24 months, 36 months, and 48 months was 7 ± 18, 14 ± 13, 15 ± 17, 17 ± 15, 22 ± 14, 23 ± 14, and 21 ± 22 ETDRS letters, respectively. During the 48 months follow-up period, the proportion of the pseudophakic eyes increased from 25% (113/460) to 67% (309/460). Long-term functional results were better for the subset of the eyes that were pseudophakic at baseline compared to the whole cohort, but the VA improvement over time was similar in both groups. The long-term visual results are shown in Figure 1.

**Figure 2:**
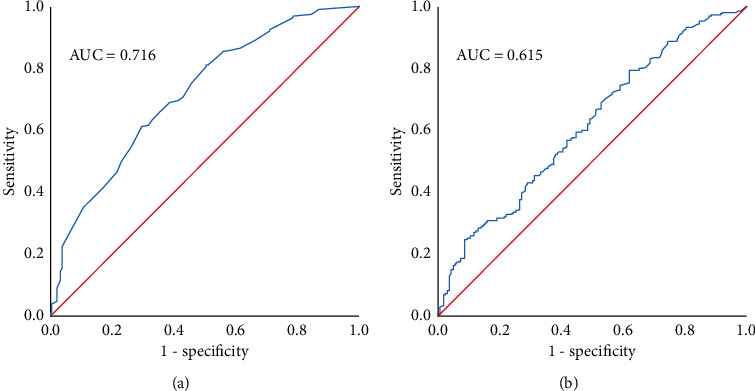
ROC curves for visual outcomes. (a) Baseline BCVA for ≥70 ETDRS letters at last follow-up. (b) MH size for VA gain ≥15 ETDRS letters between baseline and final follow-up.

ROC analysis illustrates the sensitivity and specificity of the model to predict an outcome. For the models predicting VA gain ≥15 ETDRS letters and final VA ≥70 ETDRS letters, the AUC was 0.716 and 0.615, respectively. The Youden index then designates the threshold which maximizes both sensitivity and specificity. It revealed a threshold for baseline VA of 55.5 ETDRS letters to maximize final VA ≥70 ETDRS letters and a threshold for MH size of 237 *μ*m to maximize VA gain ≥15 ETDRS letters. ROC curves are shown in Figure 2.

## 4. Discussion

While recent advances in MH surgery have improved the rate of anatomical closure, visual outcomes remain suboptimal in some patients [13–15]. Vitreoretinal surgeons have thus focused their attention on factors that may improve visual outcomes.

Several studies have previously reported that preoperative VA was the most important predictor of postoperative visual outcomes [5, 16–18]. The eyes with better baseline VA tend to obtain better postoperative final visual outcome, whereas the eyes with worse baseline VA generally gain more vision overall [16–18]. Our study is consistent with these findings. We also identified a threshold of 55.5 ETDRS letters (Snellen equivalent: 6/30) as a significant predictor of final VA ≥70 ETDRS letters.

Strengths of our study include the detailed data collection and preoperative OCT analyses, as well as the inclusion of only successful MH surgery cases to identify variables influencing postoperative VA given MH closure. Moreover, total follow-up was longer in our study (i.e., 48 months) compared to most studies with shorter follow-ups that may underestimate final visual potential [7]. Our study also differs from multicentric studies as our data come from a single hospital center, resulting in less heterogeneity in surgical practices [5, 7, 8].

An inverse correlation between MH size and postoperative vision eyes is well recognized, with larger diameter holes typically obtaining worse visual outcomes [19, 20]. While MH size was not found to be an independent predictor of final VA ≥70 ETDRS letters in our study, this was likely due to collinearity with baseline VA which was a stronger predictor.

Likewise, smaller MH size was an independent predictive factor of VA gain ≥15 ETDRS letters up to 48 months postoperative. Essex et al. identified MH size as an independent predictive factor of VA increase ≥15 ETDRS letters up to 12 months (OR: 0.88, CI: 0.79–0.99; *p*=0.037) [7]. However, in their study, routine follow-up beyond 12 months was uncommon.

Shorter duration of MH is known to be associated with better visual outcomes as there is better preservation of the macular structure and external limiting membrane (ELM) [21]. Multiple previous studies have evaluated that effect of MH on VA outcomes; these studies were limited to chronic holes and the follow-up was limited to 6 months [16, 22]. In a meta-analysis of 11 studies, the average duration of symptoms in the included studies ranged from 5.5 months to 20.5 months [23]. Another study showed that 56% (169/303) of the eyes with MH duration of symptoms shorter than 4 months had final VA ≥70 ETDRS letters at a median follow-up of 2.9 months. However, duration of symptoms was not known for 47% (499/1056) of operations in their study compared to only 4% in our study, but we did not evaluate the duration of MH using the same definition [8]. We defined MH duration as the duration between the referral date and the surgery, which helps to avoid patient recall bias with symptom onset (e.g., floaters or VA drop).

Our results suggest that proceeding quickly to surgery is associated with more favorable outcomes, especially when it is known to have benefits on postoperative VA and is the only modifiable factor unlike MH size and preoperative VA.

There is a lot of interest in identifying OCT parameters for prediction of visual outcomes. The OCT is a tool that has significantly changed the field of ophthalmology since its appearance; it is a reliable tool for monitoring retinal diseases, and various OCT biomarkers can be used clinically [24, 25]. Minimum diameter of MH (equivalent to MH size) is one of the most studied parameters. Other parameters include hole height and inner segment/outer segment junction defect length as well as ratios such as the macular hole index (MHI) [26]. In our study, we identified the presence of elevated edges on preoperative OCT as a predictive factor of final VA ≥70 ETDRS letters. A recent study by Tao et al. investigated the impact of postoperative hole edge configurations on visual outcomes and found that MH morphologies with extra flaps of tissue (*n* = 14) had significantly better final VA and postoperative restoration of ELM than MH without any specific configuration (*n* = 24) (*p*=0.012) [26]. However, to our knowledge, no studies have looked at elevated MH edge as a preoperative factor. This highlights the need to include more imaging-related preoperative variables alongside clinical data to assess visual prognosis after MH surgery.

Normal aging is associated with loss of photoreceptors and neural elements with gliosis, which may decrease the ability of the retina to restore ELM and ellipsoid zone [27]. In our study, younger age was an independent predictor factor for final VA ≥70 ETDRS letters even after excluding AMD and other ocular comorbidities that could potentially affect VA from our cohort. A few studies have found age to be a predictor, although others have not [5, 7, 8]. In the study by Essex et al., age was a predictor of VA gain of ≥15 ETDRS letters at 3 months (OR: 0.80, CI: 0.68–0.94; *p*=0.006) but not at 12 months (OR: 0.96, CI: 0.74–1.24; *p*=0.75) or 24 months (OR: 0.52, CI: 0.20–1.38; *p*=0.19) [7]. However, in our study, age was not a predictor of VA gain ≥15 ETDRS letters.

The use of TB dye was a significant predictor of final VA but not of VA gain ≥15 ETDRS letters (*p*=0.089). Better visual results with TB dye may be due to ICG toxicity on the retina. In vitro, studies using human retinal pigment epithelial cells have demonstrated the toxic effect of ICG dye and illumination [28]. ICG toxicity results in optic nerve atrophy, loss of epiretinal cellular integrity, and cellular toxicity. However, ICG dye appears to be safe when used at the clinically relevant concentration and with short time exposure, although TB showed lower toxicity [29].

MH occur most commonly in an elderly population and vitrectomy very often results in the need for cataract surgery. More than half the patients in our cohort (*n* = 194/347; 56%) were operated for cataracts over the follow-up period. Previous studies have suggested phacovitrectomy to be associated with better gain in VA and reduced health costs. Moreover, phacovitrectomy was not associated with higher rate of complications as compared to sequential vitrectomy and cataract surgery [7, 30, 31]. In our study, we included variables related to the lens status in all multiple logistic regressions to limit the effect of this confounding variable on other predictive factors.

Improvement in VA after MH surgery depends on photoreceptor restoration [32], which explains why longer follow-up is a predictor of better final VA. However, in our study, mean total follow-up time was not an independent predictive factor of VA gain ≥15 ETDRS letters.

The main limitation was the retrospective nature of the study with variable follow-up durations. Patients who had a follow-up of less than 4 weeks were excluded; these patients generally lived far from the hospital and were followed by a local ophthalmologist after their surgery. There were also several missing VA data after 24 months. However, VA mostly stabilized at 24 months, which minimizes the bias. Moreover, although the multiple logistic regression models adjusted for lens status, the size of its effect (i.e., OR = 3.948) suggests lens status still has a large effect compared to other characteristics specific to macular holes, thereby limiting the interpretation of the relative weights between lens status and the models' other variables.

Identifying the factors that predict functional outcomes in the eyes with anatomic closure may help ophthalmologists to determine factors that affect specifically visual outcomes rather than the anatomic outcomes. This may help clinicians to focus on key outcome predictors for patients and inform them accordingly. This study provides more solid evidence concerning the long-term prognostic factors. This can improve the quality of care by providing more accurate counselling to patients regarding the visual outcomes after macular hole surgery. This allows patients to make better decisions and have realistic postoperative expectations.

In conclusion, we identified preoperative VA, MH duration, and MH size as independent predictors of functional outcomes after MH surgery. We also identified preoperative VA ≥55.5 ETDRS letters (Snellen equivalent: 6/30) as a significant predictor of final VA ≥70 ETDRS letters and MH size of 237 *μ*m as a significant predictor of VA gain ≥15 ETDRS letters. Finally, future studies using big data and artificial intelligence-based methods are needed to provide a more accurate evaluation of prognostic factors of VA after MH surgery.

## Figures and Tables

**Table 1:** Baseline, intraoperative, and postoperative characteristics in the eyes with final VA ≥70 ETDRS letters versus <70 ETDRS letters following primary vitrectomy for idiopathic full-thickness macular hole.

Variables	VA ≥ 70 letters, *n* = 274	VA < 70 letters, *n* = 186	*P* value
Preoperative			
Age mean ± SD, years	67 ± 8	69 ± 8	0.145
Female gender, *n* (%)	182 (66)	134 (72)	0.223
Pseudophakia, *n* (%)	67 (25)	46 (25)	1.000
Baseline BCVA mean ± SD, letters	56 ± 12	44 ± 16	<0.001^*∗*^
MH duration mean ± SD, weeks	10 ± 6	13 ± 12	0.001^*∗*^
MH size, *μ*m (range)	314 (50–808)	415 (108–1001)	<0.001^*∗*^
MH cystic cavities, *n* (%)	259 (95)	169 (91)	1.000
MH elevated edge, *n* (%)	242 (88)	156 (84)	0.748
Operative			
Dye			0.061
ICG, *n* (%)	211 (77)	157 (84)	
TB, *n* (%)	64 (23)	30 (16)	
Tamponade			0.328
SF_6_, *n* (%)	253 (92)	167 (90)	
C_3_F_8_, *n* (%)	21 (8)	20 (11)	
Air, *n* (%)	1 (0.4)	0 (0)	
Combined phacovitrectomy, *n* (%)	1 (0.4)	1 (0.5)	1.000
Postoperative			
MH residual cystic cavities, *n* (%)	1 (0.4)	2 (1)	0.564
Phaco postvitrectomy, *n* (%)	141 (52)	53 (29)	<0.001^*∗*^
Pseudophakia at final FU visit, *n* (%)	209 (76)	100 (54)	<0.001^*∗*^
Final VA mean ± SD, letters	77 ± 4	59 ± 12	<0.001^*∗*^
Mean total FU, months	32	24	<0.001^*∗*^

BCVA, best-corrected visual acuity; SD, standard deviation; MH, macular hole; ICG, indocyanine green; TB, trypan blue; phaco postvitrectomy, phacoemulsification postvitrectomy; FU, follow-up. ^*∗*^Statistically significant.

**Table 2:** Baseline, intraoperative, and postoperative characteristics in the eyes with increase ≥15 ETDRS letters following primary vitrectomy for idiopathic full-thickness macular hole between baseline and final follow-up.

Variables	≥15 letters increase, *n* = 277	<15 letters increase, *n* = 183	*P* value
Preoperative			
Age mean ± SD, years	68 ± 8	68 ± 9	0.499
Female gender, *n* (%)	193 (70)	123 (67)	0.608
Pseudophakia, *n* (%)	67 (24)	46 (25)	0.826
Baseline BCVA mean ± SD, letters	46 ± 15	59 ± 11	<0.001^*∗*^
MH duration mean ± SD, weeks	11 ± 10	13 ± 10	0.042^*∗*^
MH size, *μ*m (range)	382 (64–1001)	313 (50–950)	<0.001^*∗*^
MH cystic cavities, *n* (%)	262 (95)	164 (90)	0.041^*∗*^
MH elevated edge, *n* (%)	246 (89)	152 (83)	0.108
Operative			
Dye			0.098
ICG, *n* (%)	213 (77)	153 (84)	
TB, *n* (%)	64 (23)	30 (16)	
Tamponade			0.412
SF_6_, *n* (%)	249 (90)	169 (92)	
C_3_F_8_, *n* (%)	27 (10)	14 (8)	
Air, *n* (%)	1 (0.4)	0 (0)	
Combined phacovitrectomy, *n* (%)	2 (0.7)	0 (0)	0.523
Postoperative			
MH residual cystic cavities, *n* (%)	1 (0.4)	2 (1)	1.000
Phaco postvitrectomy, *n* (%)	143 (52)	51 (28)	<0.001^*∗*^
Pseudophakia at final FU visit, *n* (%)	212 (77)	97 (53)	<0.001^*∗*^
Final VA mean ± SD, letters	73 ± 9	64 ± 14	<0.001^*∗*^
Mean total FU, months	30	24	<0.001^*∗*^

BCVA, best-corrected visual acuity; SD, standard deviation; MH, macular hole; ICG, indocyanine green; TB, trypan blue; phaco postvitrectomy, phacoemulsification postvitrectomy; FU, follow-up. ^*∗*^Statistically significant.

**Table 3:** Multiple logistic regression for final VA ≥70 ETDRS letters outcome.

Variables	Odds ratio	95% CI	*P* value
Age, years	0.954	0.923–0.984	0.004^*∗*^
Baseline BCVA, letters	1.064	1.040–1.091	<0.001^*∗*^
MH duration, weeks	0.950	0.915–0.983	0.005^*∗*^
MH size, *μ*m	0.999	0.997–1.000	0.140
MH elevated edge	2.721	1.099–6.688	0.029^*∗*^
TB dye	1.953	1.062–3.693	0.035^*∗*^
Pseudophakia at final FU visit	3.948	2.174–7.247	<0.001^*∗*^
Mean total FU, months	1.022	1.004–1.043	0.021^*∗*^

BCVA, best-corrected visual acuity; VA, visual acuity; CI, confidence interval; MH, macular hole; TB, trypan blue; FU, follow-up. ^*∗*^Statistically significant.

**Table 4:** Multiple logistic regression for VA increase ≥15 ETDRS letters between baseline and final follow-up^†^.

Variables	Odds ratio	95% CI	*P* value
Age, years	0.987	0.955–1.021	0.462
Baseline BCVA, letters	0.878	0.847–0.907	<0.001^*∗*^
MH duration, weeks	0.940	0.908–0.971	<0.001^*∗*^
MH size, *μ*m	0.998	0.996–0.999	0.036^*∗*^
TB dye	1.730	0.927–3.296	0.089
Preoperative pseudophakia	2.932	1.489–5.898	0.002^*∗*^
Combined phacovitrectomy or phaco-IOL postvitrectomy	4.866	2.700–8.989	<0.001^*∗*^

BCVA, best-corrected visual acuity; VA, visual acuity; CI, confidence interval; MH, macular hole; TB, trypan blue; phaco-IOL, phacoemulsification with intraocular lens implantation. ^*∗*^Statistically significant. ^†^Adjusted for bilateral disease and cystic cavities variables.

## Data Availability

The data used to support the findings of this study are available from the corresponding author upon request.
